# Reliability of a Smartphone Compared With an Inertial Sensor to Measure Shoulder Mobility: Cross-Sectional Study

**DOI:** 10.2196/13640

**Published:** 2019-09-06

**Authors:** Cristina Roldán-Jiménez, Jaime Martin-Martin, Antonio I Cuesta-Vargas

**Affiliations:** 1 Clinimetric Group F-14 Biomedical Research Institute of Malaga Malaga Spain; 2 Department of Physiotherapy Faculty of Health Sciences University of Malaga Malaga Spain; 3 Legal Medicine Area, Department of Human Anatomy, Legal Medicine and History of Science Faculty of Medicine University of Malaga Malaga Spain; 4 Institute of Health & Biomedical Innovation Faculty of Health Queensland University Technology Queensland Australia

**Keywords:** shoulder, kinematics, smartphone, mobile phone

## Abstract

**Background:**

The shoulder is one of the joints with the greatest mobility within the human body and its evaluation is complex. An assessment can be conducted using questionnaires or functional tests, and goniometry can complement the information obtained in this assessment. However, there are now validated devices that can provide more information on the realization of movement, such as inertial sensors. The cost of these devices is usually high and they are not available to all clinicians, but there are also inertial sensors that are implemented in mobile phones which are cheaper and widely available. Results from the inertial sensors integrated into mobile devices can have the same reliability as those from dedicated sensors.

**Objective:**

This study aimed to validate the use of the Nexus 4 smartphone as a measuring tool for the mobility of the humerus during shoulder movement compared with a dedicated InertiaCube3 (Intersense) sensor.

**Methods:**

A total of 43 subjects, 27 affected by shoulder pathologies and 16 asymptomatic, participated in the study. Shoulder flexion, abduction, and scaption were measured using an InertiaCube3 and a Nexus 4 smartphone, which were attached to the participants to record the results simultaneously. The interclass correlation coefficient (ICC) was calculated based on the 3 movements performed.

**Results:**

The smartphone reliably recorded the velocity values and simultaneously recorded them alongside the inertial sensor. The ICCs of the 3 gestures and for each of the axes of movement were analyzed with a 95% CI. In the abduction movement, the devices demonstrated excellent interclass reliability for the abduction humeral movement axis (Cronbach alpha=.98). The axis of abduction of the humeral showed excellent reliability for the movements of flexion (Cronbach alpha=.93) and scaption (Cronbach alpha=.98).

**Conclusions:**

Compared with the InertiaCube3, the Nexus 4 smartphone is a reliable and valid tool for recording the velocity produced in the shoulder.

## Introduction

The shoulder is one of the joints with the widest range of pathological variations, with tendonitis, bursitis, frozen shoulder, or rotator cuff involvement being some of the most common ones [1]. These pathologies cause functional alterations in the structure, which influence certain specific evaluations. Questionnaires have been developed that assess the sensitive function, pain, neuromuscular alteration, movement of structures, functionality, and mobility [2]. However, the use of these questionnaires and their validation has produced conflict [3].

Of the different questionnaires used, there is no single tool that can evaluate all the clinical aspects involved. There are several questionnaires that evaluate the performance of tasks, such as Disabilities of the Arm, Shoulder, and Hand (DASH), Western Ontario Shoulder Instability Index, and QuickDash, of which DASH is the most widely used [2]. The DASH scale also has excellent psychometric properties, with a test-retest reliability of 0.94 [4]. However, although functional tests indicate whether or not the patient is able to perform an activity, in many cases, they do not assess the range of motion directly or do not evaluate the patient’s dysfunction [5].

These questionnaires are often complemented with the use of goniometry on the assumption that this adds value to existing tests. The reliability of range-of-motion evaluations is determined by the measurement protocol used, as in the case of internal shoulder rotation [6,7]. In this respect, in addition to classical goniometry, there are systems based on digital goniometry that can be used to evaluate the range of motion with greater precision and to eliminate possible protocol deficits. However, they require specialized equipment, which is not available in many clinical situations [8]. These instruments are in many cases equipped with accelerometer-type inertial sensors [9].

Nowadays, because of the development of new technologies, the concept of telerehabilitation has emerged as an attractive method for rehabilitation at a distance, improving the quality of rehabilitation health care [10,11]. The diagnosis and assessment of musculoskeletal shoulder disorders through telerehabilitation have already been studied [12]. In this field, smartphones are well-known devices for therapeutic purposes [13], and mobile apps have transformed them into devices for clinicians [14].

Mobile phones, inertial sensors, or HALO digital goniometer have proven to be reliable instruments for the assessment of a range of motion [15,16]. These devices can be used in the clinical environment, offering savings in the costs of the assessments [17]. Devices such as the iPhone 4 have already proved to be a valid tool for measuring goniometry based on photographic measurements [18]. Smartphones are equipped with acceleration sensors that can measure variables such as speed, angulation, or acceleration [19]. These integrated sensors are accurate enough to provide angular measurements such as biofeedback in real time, if the measurement is short and the movement rate is within the effective frequency of the sensor [20]. The use of these devices in a clinical setting provides an increase in the accuracy of the record compared with visual assessments, and the same level of reliability is observed as in conventional goniometry [21].

An inertial measurement unit (IMU) sensor is one of the most reliable tools for measuring speed, acceleration, and displacement. However, there are few studies that have compared the reliability of a smartphone with that of an inertial sensor in the evaluation of patient movement [22]. The use of a smartphone’s own internal sensor has high reliability when compared with the classic goniometer in static situations [22]. To the authors’ knowledge, no additional studies have been conducted in which the reliability of a mobile device is linked to an IMU. However, smartphone devices have been used in the assessment of range of motion [17], and they are valid instruments for measuring different movements, except for the mobility of the hand. The authors of this study also mention the need to conduct validation studies in a dynamic environment.

Therefore, the aim of this study was to compare the intrasensor reliability of the measurements made by a dedicated inertial sensor with that of the measurements made by the inertial sensor integrated into a smartphone for the movements of shoulder flexion, extension, and abduction.

## Methods

### Design and Participants

This was a cross-sectional study that involved 43 subjects, of whom 16 were healthy and 27 were suffering from a shoulder injury. Asymptomatic subjects were recruited through advertisements. Patients were recruited from a specialized orthopedics clinic where they had been previously diagnosed by magnetic resonance imaging. Subjects were included if they were older than 18 years and had a body mass index (BMI) of between 18 kg/m^2^ and 42 kg/m^2^. Subjects were excluded if they refused to participate in the study. All participants were clinically examined by a physiotherapist and were interested in taking part in the project; none of them were found to meet any exclusion criteria.

Written informed consent was obtained from each individual. The study was approved by the ethics committee of the Faculty of Health Sciences at the University of Málaga, Spain.

### Data Collection and Procedures

Descriptive and anthropometric independent variables related to age, gender, weight, size, and BMI were included. The Spanish version of DASH [23] and the Upper Limb Functional Index (ULFI) [24] questionnaires were used to obtain information about shoulder disability in pathological subjects.

A physical property was included corresponding to the dependent variable of velocity (degrees per second, [º/s]). This physical property was obtained through 2 different devices.

As the criterion standard, we used an IMU with 1 inertial sensor (InertiaCube3 Intersense Inc) with dimensions 26.2 × 39.2 × 14.8 mm and weight 17 g. It contained an inertial sensor with a 3-degree-of-freedom orientation tracking system: yaw, pitch, and roll, with accuracies of 1°, 0.25°, and 25°, respectively. It also had an angular range of 360º and was able to detect an angular rate of between 0º/s and 1200º/s, with a sampling frequency of 1000 Hz. Activity values were recorded using kinematic Intersense Server Software.

The mobility angle was also measured along 3 orthogonal axes using the Nexus 4 (LG Electronics Inc) gyroscope (Invensense MPU-6050 Six-Axis [Gyro + Accelerometer]), which was attached to the posterior part of the humerus using an armband. The app used to obtain kinematic data was *Sensor Kinetics Pro* (Innoventions, Inc), which is available from Google Play. The *Nexus 4* has a storage capacity of 16 MB, and the data for each trial were transmitted by email for analysis and postprocessing. The data sampling rate was set to 14 Hz, allowing the device to record during all of the analytical tasks. Data from the smartphone and inertial sensors were subsequently passed to a Microsoft Excel 2007 database.

The inertial sensor was placed on the side of the body of each subject on which the shoulder presented pathology and was located on the middle third of the humerus, slightly to the posterior. Its surface was cleaned with alcohol to allow the sensor to adhere to the skin. To ensure fixation of the sensor to the patient’s skin and to prevent slippage, a double-sided adhesive tape and an 8-cm-wide elastic cohesive (Rapidex) were used.

The smartphone was placed slightly below the inertial sensor and was snugly secured by a neoprene fixation belt over the humerus. The orientation and movement of the sensors are presented as roll, pitch, and yaw Euler angles. The equivalence of the axes on both devices and their anatomical interpretations are shown in Table 1.

After participants were recruited for the study, they were asked to attend the Human Movement Laboratory, Faculty of Health Sciences (University of Málaga). The tasks were explained concisely and clearly so that each participant understood the action to be performed. The beginning and end were determined by a verbal order by the researcher. Participants were instructed to stand; starting from a neutral position, they were asked to perform the following analytical tasks:

1. Shoulder abduction, with the elbow extended, wrist in a neutral position, and the palmar area of the hand toward the midline at the beginning and end of the movement (4 repetitions)
2. Shoulder flexion, with the elbow extended, wrist in a neutral position, and the palmar area of the hand toward the midline at the beginning and end of the movement (4 repetitions)
3. Shoulder scaption, with the elbow extended, wrist in a neutral position, and the palmar area of the hand toward the midline at the beginning and end of the movement (4 repetitions).

### Data Processing

**Figure 1:**
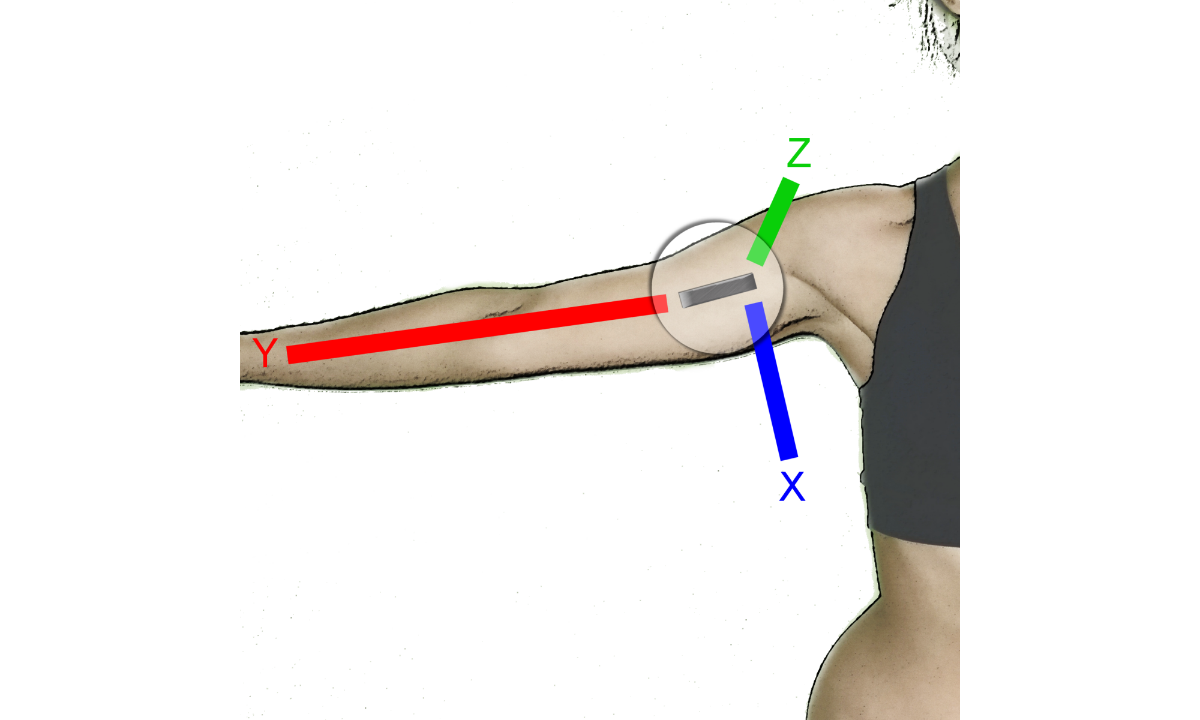
X, Y, and Z motion axes.

A computerized automatic analysis was conducted to filter the inertial sensor data. This analysis, which was designed to systematically obtain kinematic data for further statistical analysis, was performed using the basic software package R. As the sampling frequency of the Nexus 4 was 14 Hz and the frequency of the InertiaCube3 was 1000 Hz, the data were resampled to equalize the sampling frequencies at 100 Hz. Likewise, a common time 0 was established for all measurements made based on the time units obtained by the sensors. The automatic analysis was guided to obtain kinematic information from the accelerometer and gyroscope independently for each subject. The means and SDs of velocity in the 3 axes of movements (X, Y, and Z) were obtained from the accelerometer. The sign of the measured values of the accelerometer velocity along the X, Y, and Z axes is shown in Figure 1.

### Statistical Analysis

A descriptive statistical analysis was conducted based on the means and SDs of the values characterizing the sample (age, weight, height, BMI, ULFI 100, and DASH 100). An interclass correlation analysis was performed between the variables recorded by the smartphone and the IMU. The results were analyzed for the 3 different gestures made by the participants (abduction, flexion, and scaption), and for each of these movements, the yaw, pitch, and roll axes were compared. The reliabilities of the mobile device and the IMU were estimated by means of the interclass correlation coefficient (ICC) and their 95% CIs based on a 2-way mixed model and absolute type using the SPSS statistical package version 22 [25]. The ICC value for a single measure is an index of the reliability of the ratings for 1 typical single rater (intrarater reliability). However, the average measure of the ICC is an index for the reliability of different rates averaged together (interrater reliability). This ICC is always higher than the single measurements of the ICC [26]. Values less than 0.5 are indicative of poor reliability, whereas values between 0.5 and 0.75 indicate moderate reliability, values between 0.75 and 0.9 indicate good reliability, and values greater than 0.90 indicate excellent reliability [27].

**Table 1:** Equivalence between the inertial sensor and smartphone placed on the humerus and the anatomical interpretation for shoulder movements.

Euler angles	Sensor axes	Smartphone axes	Anatomical axes	Anatomical planes	Shoulder movements
Yaw	Yaw	Z	Anteroposterior (dorsoventral)	Coronal (frontal)	Abduction
Pitch	Pitch	Y	Craniocaudal	Transverse (horizontal)	Rotation
Roll	Roll	X	Left-right	Midsagittal (median)	Flexoextension

### Ethical Aspects

This study meets the criteria established by the Helsinki Declaration, and all participants were informed and signed an informed consent statement. The data obtained were treated anonymously. Ethical endorsement was obtained from the research committee of the Faculty of Health Sciences of the University of Málaga.

## Results

The sample included 16 asymptomatic (controls) and 27 pathological subjects (patient) (7 with subacromial syndromes, 6 with supraspinatus tendon rupture, 9 with rotator cuff tear, 3 with supraspinatus tendinopathy, 1 with shoulder instability, and 1 with a Superior Labrum Anterior to Posterior lesion). The characteristics of the population and the results for shoulder functionality are shown in Table 2.

### Shoulder Abduction

The ICC for the abduction shoulder movement was excellent for the abduction humeral movement (Table 3). This axis is related to the movement in the coronal plane, which directly determines the abduction movements (Table 1). The rotation and flexoextension movements of the humerus were moderate.

### Shoulder Flexion

The ICC values for the flexion shoulder movement were excellent in terms of the abduction humeral movement between both the devices, and this was true for both the individual measurements and the averages of the measurements. In the same way, for the flexoextension humeral movement (left-right axes), the ICC was very good, both for the mean measures and single measures (Table 4).

### Shoulder Scaption

For the scaption shoulder movement, excellent results were observed for the ICC for the abduction humeral movement (Table 5), and a very good ICC was recorded for the movements of flexion and humeral extension.

**Table 2:** Characteristics of participants.

Descriptive variable	Patients (n=27)	Controls (n=16)
Age (years), mean (SD)	52.8 (9.8)	55.6 (8.9)
Weight (kg), mean (SD)	77.1 (18.3)	73.7 (14.1)
Height (m), mean (SD)	1.6 (0.1)	1.6 (0.1)
Body mass index (kg/m^2^), mean (SD)	28.4 (6.7)	26.9 (3.6)
Upper Limb Functional Index, % mean (SD)	70.1 (24.5)	0 (0)
Disabilities of the Arm, Shoulder and Hand, % mean (SD)	63.2 (20.4)	0 (0)

**Table 3:** Interclass correlation coefficient interdevices for abduction movement.

Humerus movement and measure		Interclass correlation coefficient (95% CI)	Cronbach alpha
**Abduction**			.980
	Single	0.947 (0.841-0.978)	
	Mean	0.973 (0.913-0.989)	
**Rotation**			.610
	Single	0.435 (0.130-0.666)	
	Mean	0.606 (0.230-0.800)	
**Flexoextension**			.656
	Single	0.479 (0.186-0.696)	
	Mean	0.648 (0.314-0.821)	

**Table 4:** Interclass correlation coefficient interdevices for flexion movement.

Humerus movement and measure		Interclass correlation coefficient (95% CI)	Cronbach alpha
**Abduction**			.925
	Single	0.855 (0.733-0.924)	
	Mean	0.922 (0.846-0.960)	
**Rotation**			.616
	Single	0.444 (0.143-0.670)	
	Mean	0.615 (0.251-0.803)	
**Flexoextension**			.876
	Single	0.770 (0.593-0.876)	
	Mean	0.870 (0.745-0.934)	

**Table 5:** Interclass correlation coefficient interdevices for scaption movement.

Humerus movement and measure		Interclass correlation coefficient (95% CI)	Cronbach alpha
**Abduction**			.948
	Single	0.896 (0.795-0.949)	
	Mean	0.945 (0.886-0.974)	
**Rotation**			
	Single	0.427 (0.101-0.673)	.606
	Mean	0.598 (0.184-0.804)	
**Flexoextension**			.824
	Single	0.673 (0.413-0.830)	
	Mean	0.805 (0.584-0.907)	

## Discussion

### Principal Findings

This study validated the use of a smartphone as an instrument to assess arm velocity during shoulder flexion, abduction, and scaption. An IMU was used as a reference system, and it proved to be an excellent, reliable, cheap, and easy-to-use tool for the measurement of humeral velocity. This approach would allow kinematic information to be transferred to a clinical context.

For the abduction movement (Table 3), the devices demonstrated excellent interclass reliability on the abduction humeral movement axis (alpha=.98); however, these values were not replicated in the rotation and flexoextension humeral movements. The same results were observed when both mean values and individual measurements were analyzed.

The intersensor reliability was excellent for the movements of flexion and scaption in abduction humeral movement, with ICC=0.925 and ICC=0.984, respectively (Tables 4 and 5). However, for the rotation and flexoextension humeral movement, the results ranged between moderate and good. The reliability of the rotational humeral movement was moderate for the 3 movements analyzed (Tables 3-5). The agreement between the measurements made by the devices was good for the movements of flexion and scaption on the flexoextension humeral movement (Tables 4 and 5).

The lower reliability for the different axes of movement can be related to the compensation for the movement that is being made; this implies a modification of the speed of movement, which prevents an adequate record being made by the Nexus 4. Mourcou et al [22] observed that the Nexus 4 smartphone did not have the same reliability when analyzing the movement on the roll axis compared with the pitch axis. When the smartphone is in a static position, it has good roll and pitch reliability with an error of 0.2°, without significant differences; however, when there is an increase in velocity, the smartphone’s detection algorithms have more difficulty in identifying and following the movement [22]. Likewise, the filter applied to the data also influences the variation in the recorded data, and the filter itself produces the most variation in the measurements.

Other studies have analyzed the reliability of mobile devices as clinical tools compared with an inertial sensor, in a similar way [22,28]. Pichonnaz et al validated the use of a smartphone (iPod, Apple) with an ICC of alpha=.97 compared with a Physilog reference system (Gait Up) using the B-B score test (hand to the Back and hand upwards as to change a Bulb) [29]. However, their study did not analyze the reliability of the isolated smartphone for each of the movements as in this study.

The inertial sensors included in smartphones can be taken as valid biofeedback if the variations in the acceleration are in the range ±8 g with an angular velocity of ±2000º /s and a sampling frequency of 100 Hz [30]. To these factors, we must add the smartphone’s own error in the accelerometer record of ±40 mg and the error produced by the gyroscope of ±1°/s, which cannot be compensated for in the original data [15]. The lowest levels of reliability were observed in the rotational humeral movement relative to the corresponding craniocaudal axis (rotation abduction ICC=0.610, rotation flexion ICC=0.616, and rotation scaption ICC=0.606, as shown in Tables 3-5, respectively). These levels may be related to the internal limitations of the inertial sensor and to possible compensations made by the patients during the movements that are linked to the mobility of the scapula and that have a direct impact on the glenohumeral joint [31].

There are some inherent limitations of the method used in this study. First, the smartphone used was a Nexus 4, which is a relatively old model in terms of its inertial sensor; other, more current inertial sensors included in mobile devices may be more reliable. No additional filters were applied to the recorded data to keep the record intact in the same way as a clinician would, and this may imply a limitation on its reliability. In this study, only the humerus was evaluated, without assessing the possible implications that the scapula could have in terms of providing additional reliability or correlation. However, the application of our protocol was positive in both patients and healthy subjects. In the same way, the implementation environment was close to the clinical reality, and the use of a common mobile device fixed to the patient does not involve excessive equipment. There are few studies in which the reliability of a mobile device is evaluated in comparison with a reference system.

### Conclusions

The objective of this study was to validate the Nexus 4 as a tool for the analysis of shoulder movement. Compared with an IMU, the inertial sensor included in the Nexus 4 smartphone proved to be a tool with excellent reliability for intersensor mediation in the velocities produced in the humerus during shoulder flexion, abduction, and scaption movements in the yaw axis. Its reliability is good when measurements are made on the pitch axis, which is linked to the left-right axis. To increase the reliability of the device, both the velocity of movement and the possible deviations or compensations that may appear must be controlled.

## Abbreviations

BMI: body mass index
DASH: Disabilities of the Arm, Shoulder, and Hand
ICC: interclass correlation coefficient
IMU: inertial measurement unit
ULFI: Upper Limb Functional Index
